# *N*-Desmethyldauricine Induces Autophagic Cell Death in Apoptosis-Defective Cells via Ca^2+^ Mobilization

**DOI:** 10.3389/fphar.2017.00388

**Published:** 2017-06-16

**Authors:** Betty Y. K. Law, Simon W. F. Mok, Juan Chen, Francesco Michelangeli, Zhi-Hong Jiang, Yu Han, Yuan Q. Qu, Alena C. L. Qiu, Su-Wei Xu, Wei-Wei Xue, Xiao-Jun Yao, Jia Y. Gao, Masood-ul-Hassan Javed, Paolo Coghi, Liang Liu, Vincent K. W. Wong

**Affiliations:** ^1^State Key Laboratory of Quality Research in Chinese Medicine, Macau University of Science and TechnologyMacau, China; ^2^The Key Laboratory of Molecular Biology on Infectious Diseases, Ministry of Education, The Second Affiliated Hospital of Chongqing Medical University, Chongqing Medical UniversityChongqing, China; ^3^Department of Biological Sciences, University of ChesterChester, United Kingdom; ^4^College of Chemistry and Chemical Engineering, Lanzhou UniversityLanzhou, China; ^5^College of Medicine, King Saud Bin Abdulaziz University for Health SciencesJeddah, Saudi Arabia

**Keywords:** *N*-desmethyldauricine, SERCA, autophagy, autophagic cell death, apoptosis-resistant

## Abstract

Resistance of cancer cells to chemotherapy remains a significant problem in oncology. Mechanisms regulating programmed cell death, including apoptosis, autophagy or necrosis, in the treatment of cancers have been extensively investigated over the last few decades. Autophagy is now emerging as an important pathway in regulating cell death or survival in cancer therapy. Recent studies demonstrated variety of natural small-molecules could induce autophagic cell death in apoptosis-resistant cancer cells, therefore, discovery of novel autophagic enhancers from natural products could be a promising strategy for treatment of chemotherapy-resistant cancer. By computational virtual docking analysis, biochemical assays, and advanced live-cell imaging techniques, we have identified *N*-desmethyldauricine (LP-4), isolated from *rhizoma of Menispermum dauricum DC* as a novel inducer of autophagy. LP-4 was shown to induce autophagy via the Ulk-1-PERK and Ca^2+^/Calmodulin-dependent protein kinase kinase β (CaMKKβ)-AMPK-mTOR signaling cascades, via mobilizing calcium release through inhibition of SERCA, and importantly, lead to autophagic cell death in a panel of cancer cells, apoptosis-defective and apoptosis-resistant cells. Taken together, this study provides detailed insights into the cytotoxic mechanism of a novel autophagic compound that targeting the apoptosis resistant cancer cells, and new implication on drug discovery from natural products for drug resistant cancer therapy.

## Introduction

Autophagy is a cellular degradation mechanism characterized by the formation of double membrane cytoplasmic vesicles, which engulf and degrade cytoplasmic organelles such as mitochondria or ER through lysosomes, thereby, regulate normal cellular integrity and homeostasis of cells. Genetic deletion of Atg has revealed the roles of autophagy in response to cellular differentiation, development, homeostasis, starvation, and stressful conditions (Levine and Kroemer, 2008). Mice models with deletion of Atg genes has revealed the correlation between autophagy and diseases including neurodegenerative diseases, infectious diseases, metabolic diseases, and cancers (Jiang and Mizushima, 2014). In cancers therapies, autophagy can act as either a tumor suppressor by the removal of damaged proteins and organelles, or as a pro-survival mechanism to promote the pathogenesis of tumors (Yang et al., 2011). For example, constitutive activation of autophagy could eventually lead to autophagic cell death (type II programmed cell death) (Tsujimoto and Shimizu, 2005). Monoallelic loss of the essential autophagy gene, beclin 1, were found in human breast, prostate, and ovarian cancers, suggesting the role of autophagy in preventing tumorigenesis (Yang et al., 2011). However, resistant to apoptosis remains a major obstacle in cancer therapies. Emerging evidence have reported novel compounds such as polyphenolic natural compounds curcumin, rottlerin, quercetin, genistein and resveratrol (Hasima and Ozpolat, 2014), STF-62247 (Turcotte et al., 2008) and guttiferone K (Wu et al., 2015) are capable of regulating cancers via the autophagic cell death mechanism (Bursch et al., 1996; Maiuri et al., 2007). Although clinically approved agents such as rapamycin, plays a therapeutic role in cancer therapy (Opipari et al., 2004; Hoyer-Hansen et al., 2005; Kondo et al., 2005; Chang et al., 2007; Law et al., 2010, 2014; Yang et al., 2011; Wong et al., 2013), mTOR inhibition has adverse effects in protein synthesis, cell proliferation, and immune function (Levine and Kroemer, 2008; Pallet and Legendre, 2013). Therefore, drugs that can enhance autophagic cell death, especially in apoptosis-resistant cells, with minimal side effects would be highly desirable.

Our previous findings have identified a group of natural alkaloid small-molecules, including liensinine, isoliensinine, dauricine, and cepharanthine, which stimulated the induction of autophagy and autophagic cell death in a panel of apoptosis-resistant cells (Law et al., 2014). Dauricine, the major bioactive alkaloid isolated from *Menispermum dauricum* D.C. (Jin et al., 2010), has been widely prescribed to treat inflammatory diseases (Yang et al., 2010), allergy, and arrhythmia in the local Chinese community. The reported pharmacological effect of dauricine has been attributed to its anti-arrhythmic effect and the ability to modulate Ca^2+^ and several K^+^ channels. (Zhao et al., 2012). Based on spectrometric analysis and *N*-methylation method which offered the derivative of (dauricine dimethiodide), *N*-desmethyldauricine (LP-4) were firstly isolated from nature in Pan (1992) with unknown biological effects.

It was reported that cell differentiation, contraction of muscle, gene transcription and cell death are highly regulated by the change in cytosolic calcium level (Berridge et al., 2000). Although the role of calcium regulating autophagy remains controversial, several literatures reported the calcium mobilizing agents such as alisol B, thapsigargin, ATP, vitamin D3 and ionomycin activated autophagy via the calcium-activated kinase (CAMKK)-β-AMPK- mTOR pathway (Hoyer-Hansen et al., 2007; Law et al., 2010). With the critical role of calcium involved in cellular signaling pathways responsible for tumorigenesis, alternating the homeostasis of calcium lead to cancers (Monteith et al., 2007, 2012; Pereira et al., 2011), therefore, the potential role of calcium-regulated autophagy in modulating pathogenesis of cancers worth our further investigation. Upon cellular stressful conditions such as deprivation of nutrient, infection, accumulation of unfolded or misfolded proteins, stimulation by hypoxia, toxins or oxidative injury, and aberrant regulation on calcium level, UPR will be triggered to restore the normal function of ER. Autophagy has been emerged as an important cellular protective mechanism during ER stress (Rashid et al., 2015).

In our current study, we report for the first time that LP-4, inhibits SERCA, leading to calcium release and induction of autophagy via the ULK and CaMKK-β-AMPK-mammalian target of rapamycin (mTOR)-dependent pathway. As one of the key mechanistic pathway triggering the induction of autophagy, we show that LP-4 causes Ca^2+^ release in cells and induces the UPR. By computational virtual docking analysis and biochemical assays, we demonstrate that LP-4 inhibits SERCA in a dose dependent manner which is co-incident with the concentrations leading to autophagic cell death in a panel of cancer cells, apoptosis-defective, and apoptosis-resistant cells. Our study provides pharmacological insights into the protective mechanism of LP-4 in its potential anti-cancer therapeutic application, and proposes a new direction of identifying novel autophagic inducers from natural products as a new therapeutic perspective for treating apoptosis-resistant cancers.

## Materials and Methods

### Antibodies, Plasmids, Chemicals, and Reagents

Unless otherwise specified, all chemicals and reagents were obtained from Sigma–Aldrich. BAPTA/AM (BM), compound C (CC), E64D, pepstatin A, thapsigargin, inositol trisphosphate, STO-609 and AMD3100 were purchased from Calbiochem (San Diego, CA, United States). *N*-desmethyldauricine (>98% purity, HPLC) were purchased from the China Chengdu Biotechnology Company Ltd (Chengdu, China). Antibodies against AMPK, phospho-AMPKα (Thr172), p70S6 kinase, phospho-p70S6 kinase (Thr389), LC3B, PERK, eIF2α and phospho-eIF2α (Ser51) were purchased from Cell Signaling Technologies Inc. (Beverly, MA, United States). Antibodies against CXCR4, IgF-1, p62, and ULK-1 were obtained from Santa Cruz Biotechnology (Santa Cruz, CA, United States). Antibodies against beta-actin were obtained from Sigma (St. Louis, MO, United States). Antibodies against phospho-PERK (Thr980) were purchased from BioLegend (San Diego, CA, United States). TRITC-conjugated anti-mouse secondary antibodies (ZyMax^TM^) were purchased from Invitrogen (Scotland, United Kingdom). siRNAs targeting IgF-1, ULK-1, PERK, and non-silencing negative control siRNA (AllStars) were obtained from Qiagen (Hilden, Germany). pEGFP-LC3 reporter plasmid was provided by Prof. Tamotsu Yoshimori (Osaka University, Japan).

### Cell Culture

Unless otherwise specified, all cells were obtained from the American Type Culture Collection, ATCC (Rockville, MD, United States). Caspase 3/7-deficient and wild-type MEFs were generous gift provided by Prof. Richard A. Flavell (Yale University School of Medicine, United States). Bax-Bak double knockout MEFs were provided by Prof. Shigeomi Shimizu (Tokyo Medical and Dental University, Medical Research Institute, Japan). Caspase 8-deficient MEFs were generous gift from Prof. Kazuhiro Sakamaki (Kyoto University, Graduate School of Biostudies, Japan). Both Atg7 wild-type and deficient MEFs were gifts from Prof. Masaaki Komatsu (Juntendo University, School of Medicine, Japan). All cells were maintained in culture medium supplemented with 10% fetal bovine serum, penicillin (50 U/ml) and streptomycin (50 μg/ml) (Invitrogen, Paisley, Scotland, United Kingdom) at 37°C with 5% CO_2_.

### Quantification of Green Fluorescent (GFP) LC3 Puncta Formation

Cells transfected with EGFP-LC3 were treated with LP-4 (10 μM) and then fixed with 4% of paraformaldehyde (Sigma). The formation of EGFP-LC3 puncta was exanimated and quantitated by fluorescent microscopic analysis (Applied Precision DeltaVision Elite, Applied Precision, Inc., United States) following autophagy guidelines (Klionsky et al., 2016). In brief, the number of cells with increased EGFP-LC3 fluorescence puncta (≥10 dots/cell) over the total number of EGFP-positive cells were calculated. For each experiment, a minimum of 1000 cells from randomly selected fields were scored.

### Detection of Cytotoxicity and Apoptosis

Cell cytotoxicity assay was used to measure cell viability (IC_50_ value). In brief, the percentage of cell viability was calculated as: Cells number_treated_/Cells number_DMSO control_ × 100. Annexin V (BD Biosciences, San Diego, CA, United States) stained cells were measured by FACSAria III flow cytometer (BD Biosciences) to detect apoptosis. Data obtained from three independent experiments were analyzed by using CellQuest (BD Biosciences).

### Transmission Electron Microscopy

In brief, cells were first fixed overnight with 2.5% of glutaraldehyde, and then post-fixed in 1% of OsO4 before embedded in Araldite 502 for microscopy. Ultrathin sections stained with uranyl acetate and lead citrate were then analyzed by transmission electron microscope (Philips CM100) at a voltage of 80 kV.

### PCR Array Analysis

Total RNA were obtained by using Qiagen RNeasy^®^ Mini Kit (Qiagen). cDNA was synthesized by performing reverse transcription using RT^2^ first strand kit (Qiagen). The human autophagy pathway specific RT^2^ Profiler PCR array (Qiagen), comprises of 87 autophagy related genes involved in regulating autophagy, was used to evaluate the potential mechanistic pathways of LP-4 in HeLa cells. Real-time PCR reactions were performed by using RT^2^ SYBR^®^ Green qPCR Mastermix (Qiagen) with the ViiA^TM^ 7 Real Time PCR System (Applied Biosystems). Integrated web-based software package (Qiagen) which calculated all ΔΔCt based fold-change from threshold cycle raw data was used for data analysis.

### Intracellular Free Calcium Measurement

HeLa cells after treatment of LP-4 (5 or 10 μM) were washed twice with MEM. The cell suspensions were then stained with 5 μM of Fluo-3, a high sensitive fluorescent dye for rapid measurement of calcium flux in cells, at 37°C for 30 min. The cells were washed twice with HBSS before subjected to FACS analysis with at least 10,000 events measured.

### Measurement of Cytoplasmic Calcium Dynamic

Intracellular cytosolic Ca^2+^ dynamic was measured using the FLIPR Calcium 6 Assay Kit (Molecular Devices) according to the manufacturer’s instructions. In brief, HeLa cells were plated at a density of 10000 cells per well in black wall/clear bottom 96-multiwell plates from Costar (Tewksbury, MA, United States) and cultured for 24 h before treatment. After that, calcium 6 reagent was added directly to cells, and cells were incubated for an additional 2 h at 37°C and 5% CO_2_. 5 and 10 μM of LP-4 were then added to the wells and immediately subjected to data acquisition on the FLIPR Tetra High-Throughput Cellular Screening System (Molecular Devices) at room temperature using a 1-s reading interval throughout the experiments.

### Single Cell Calcium Imaging

2 × 10^5^ HeLa cells were cultured in 35 mm confocal disk at 37°C CO_2_ incubator for 24 h. 5 mM of Fluo 3/AM/DMSO stock solution was diluted to 5 μM working solution using Hanks-balanced salt solution (HBSS) and then added to cells at 37°C for 30 min. HeLa cells were then washed three times with HEPES buffer saline and incubated at 37°C in an imaging chamber for another 10 min. Changes in cytosolic [Ca^2+^] levels were monitored by following changes in fluo-3 fluorescence upon addition of 10 μM LP-4 in HBSS buffer, using the real-time mode for 5 min by epifluorescence microscopy (Applied Precision DeltaVision Elite, Applied Precision, Inc., United States). Data Inspection Program provided by the DeltaVision software was used to measure the intensity of the fluo-3 fluorescence and the mean fluorescence intensity was monitored at 523 nm and plotted against time (sec).

### Computational Docking

The initial 3D structures for LP-4 were downloaded from the PubChem^1^. Then, the inhibitors were preprocessed by the LigPrep which uses OPLS-2005 force field and gave the corresponding low energy conformers of the compounds. The ionized state was assigned by using Epik at a target pH value of 7.0 ± 2.0. The co-crystal structure of sarco/endoplasmic reticulum Ca^2+^ ATPase (SERCA) complexed with thapsigargin (TG) was retrieved from the Protein Data Bank [PDB ID code 2AGV (Obara et al., 2005)]. To prepare the protein for docking, the Protein Preparation Wizard module in Schrödinger 2009 was used to remove crystallographic water molecules, add hydrogen atoms, assign partial charges using the OPLS-2005 force field, assign protonation states, and minimize the structure. The minimization was terminated when the root-mean-square deviation (RMSD) reached a maximum value of 0.30 Å. In molecular docking, the prepared LP-4 was docked into the TG binding site of the SERCA using the Glide with the extra precision (XP) scoring mode. The docking grid box was defined using the Receptor Grid Generation tool in Glide by centering on TG in the SERCA. In molecular docking, 5000 poses were generated during the initial phase of the docking calculation, out of which best 1000 poses were chosen for energy minimization by 1000 steps of conjugate gradient minimizations. The best binding pose for LP-4 was considered for the further analysis.

### Measurement of SERCA Activity

The activity of Ca^2+^ ATPase (SERCA1A) purified from female rabbit hind leg muscle (Michelangeli and Munkonge, 1991) was measured by using the enzymatic method utilizing pyruvate kinase and lactate dehydrogenase as described previously (Michelangeli et al., 1990). In brief, all SERCA inhibition data were fitted to the allosteric dose vs. effect equation using Fig P (Biosoft, Cambridge, United Kingdom): Activity = minimum activity + (maximum activity - minimum activity)/(1 + ([I]/IC_50_)^P^).

### Live-Cell Imaging

After treatment with LP-4, the induction of autophagy was monitored in EGFP-LC3 transfected cells at 37°C supplied with 5% of CO_2_. Treated cells were then observed under oil objective (60× Olympus PlanApoN 1.42) at a wavelength of 512 nm. Under high magnification wide field epifluorescence microscopic analysis, DIC and fluorescent images were captured at 5-min intervals. Images were captured as serial Z-sections (1.0 μm interval) by using Olympus IX71-Applied Precision DeltaVision restoration microscope (Applied Precision, Inc., United States) equipped with Photometrics CoolSNAP HQ^2^ CCD camera. The epifluorescence images were numerically deconvolved by using DeltaVision algorithms (Applied Precision, Inc.).

### Statistical Analysis

The results were expressed as means ± SD as indicated. The differences were considered statistically significant when the *P*-value was less than 0.05. Student’s *t*-test or one-way ANOVA analysis was used for comparison among different groups.

## Results

### LP-4 Induces Autophagy and Cell Death toward Different Cancer Cell Lines

**FIGURE 1:**
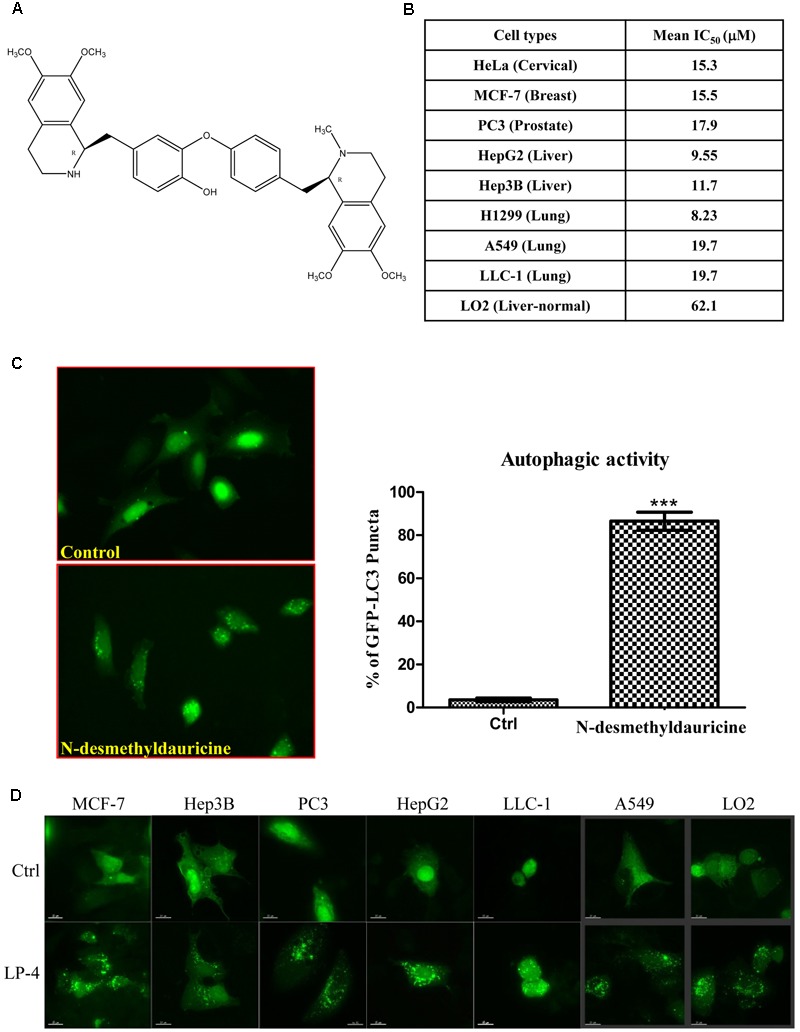
Autophagic activity of *N*-desmethyldauricine (LP-4). **(A)** Chemical structure of *N*-desmethyldauricine. **(B)** Cytotoxicity (IC_50_ value) of LP-4 on a panel of normal and cancerous cell line from different origins. **(C)** Detection of EGFP-LC3 in LP-4 treated cells. HeLa cells transfected with the EGFP-LC3 plasmid for 24 h were treated with DMSO (Control) or LP-4 (10 μM) for 4 h. Representative images showing the formation of EGFP-LC3 puncta were captured. Autophagic activity was defined by the number of cells with increased EGFP-LC3 fluorescence puncta (≥10 dots/cell) over the total number of EGFP-positive cells. Approximately 1000 EGFP-positive cells were scored for each treatment. Data were represented as the means of three independent experiments; error bars, SD ^∗∗∗^*P* < 0.001. **(D)** The detection of LP-4 induced autophagy in both cancerous and normal cells. A panel of cancer cells including MCF-7, Hep3B, PC3, HepG2, LLC-1, A549 and normal liver cells (LO2) transfected with the EGFP-LC3 plasmid for 24 h were treated with LP-4 (10 μM) for 4 h. Representative images were captured (60× magnification). Scale bar, 15 μm.

Cytotoxicity of LP-4 (**Figure 1A**) was measured against cancer cells of different origins, including HeLa, MCF-7, PC3, HepG2, Hep3B, H1299, A549, LLC-1, and normal human hepatocytes (LO2). As shown in **Figure 1B**, LP-4 was more toxic in HepG2 liver cancer and H1299 lung cancer cells (mean IC_50_ < 10 μM). However, the cytotoxicity of LP-4 was much lower in LO2 normal liver hepatocytes (mean IC_50_ = 62.1 μM), suggesting that LP-4 exhibited specific cytotoxic effects toward cancer cells.

**FIGURE 2:**
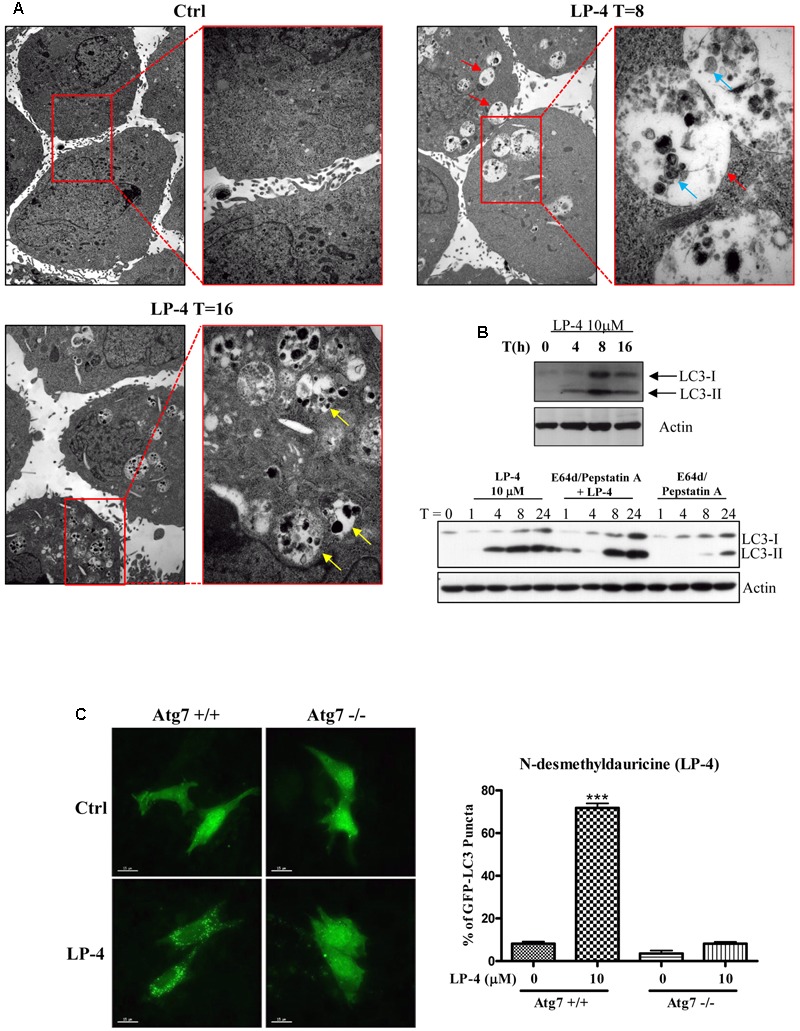
*N*-desmethyldauricine (LP-4) induces autophagy through an Atg7 dependent mechanism. **(A)** LP-4 induced the formation of autophagosomes/autolysosome in cells. After the treatment of LP-4 (10 μM), the changes of the ultra-structures of HeLa cells were captured and showed as electron micrographs (40000× magnification). The highlighted red regions represented the magnified images of the double-membraned autophagosomes (red arrow), autolysosomes (yellow arrow) and engulfed organelles (blue arrow), respectively. **(B)** LP-4 induced autophagic flux. HeLa cells treated with LP-4 (10 μM) in the presence or absence of 10 μg/mL lysosomal protease inhibitors (E64d and Pepstatin A) for 24 h. Cell lysates were analyzed by western blot for LC3-II conversion (LC3-I, 18 kDa; LC3-II, 16 kDa), and actin, respectively. **(C)** LP-4 induced autophagy through an Atg7 dependent mechanism. Both Atg7 -wild-type (Atg7^+/+^) and -deficient (Atg7^-/-^) MEFs transfected with EGFP-LC3 plasmid were treated with LP-4 (10 μM) for 4 h. Autophagic activity was evaluated as the number of cells with increased EGFP-LC3 fluorescence puncta (≥10 dots/cell) over the total number of EGFP-positive cells. Data was represented as the means of three independent experiments; scale bar, 15 μm; error bars, SD ^∗∗∗^*P* < 0.001.

The induction of autophagy may lead to autophagic cell death in some apoptosis-resistant cancers through the inhibition of anti-autophagic proteins (Dalby et al., 2010), thus, identification of novel autophagy inducers from natural products may act as an effective strategy for the discovery of anti-cancer compounds (Turcotte and Giaccia, 2010). To evaluate the autophagic effect of LP-4, the conversion of cytosolic LC3-I to membrane-bound LC3-II, an essential step for the induction of autophagy, was monitored by transiently expressing HeLa cells with GFP-LC3 protein (Kuma et al., 2007; Tanida et al., 2008). As revealed by the increased formation of GFP-LC3 puncta in HeLa cells, the result indicated that LP-4 could significantly induce autophagy (**Figure 1C**). To determine whether LP-4 could induce autophagy in other cancer and normal cell types, MCF-7, Hep3B, PC3, HepG2, LLC-1, A549 and normal human hepatocytes, LO2 were used. As shown in **Figure 1D**, LP-4 induced GFP-LC3 puncta formation in both normal and cancer cells, suggesting that the autophagic effect of LP-4 is not cell types specific. We further analyzed the ultra-structures of HeLa cells treated with LP-4 using transmission electron microscopy. As shown in **Figure 2A**, the number of double-membrane autophagosomes increased in a time-dependent manner in response to LP-4 treatments. Autophagic vacuoles (autolysosomes) with engulfed organelles were also identified in cells treated with LP-4 for 16 h (**Figure 2A**). As autophagosome accumulation could result from either an induction of autophagic flux or the blockage of fusion between autophagosome and lysosome (Mizushima and Yoshimori, 2007; Levine and Kroemer, 2008), we measured the formation of LC3-II in the presence of lysosomal protease inhibitors (E64d and pepstatin A) (Law et al., 2014). As shown in **Figure 2B**, LP-4 increased the rate of LC3-II formation in the presence of the protease inhibitors when compared with the addition of either protease inhibitors or LP-4 alone. These findings confirmed that LP-4 induced autophagy as a result of increased formation of autophagosome.

### LP-4 Induces Autophagy Dependent on Autophagy-Related Gene (Atg) 7

The elongation of the autophagosomal membrane is highly regulated by the ubiquitin-like conjugation systems (Ohsumi and Mizushima, 2004). For example, the conjugation of Atg12 to Atg5 requires the ubiquitin-activating-enzyme-like Atg7 and Atg10 (Juenemann and Reits, 2012), which are essential for autophagic vesicle nucleation and elongation (Levine and Kroemer, 2008). To study the role of Atg7 in LP-4-induced autophagy, we over-expressed the GFP-LC3 plasmids in both Atg7 wild-type and deficient MEFs. Results indicated that LP-4 induced the formation of GFP-LC3 puncta in Atg7 wild-type MEFs, the percentage of cells with GFP-LC3 puncta formation was very low in Atg7 deficient MEFs, which are resistant to autophagy induction (**Figure 2C**). This result indicated the involvement of Atg7 in LP-4-mediated induction of autophagy.

### LP-4 Induces Autophagy through Up-regulation of ULK-1 and PERK Gene Expression

**FIGURE 3:**
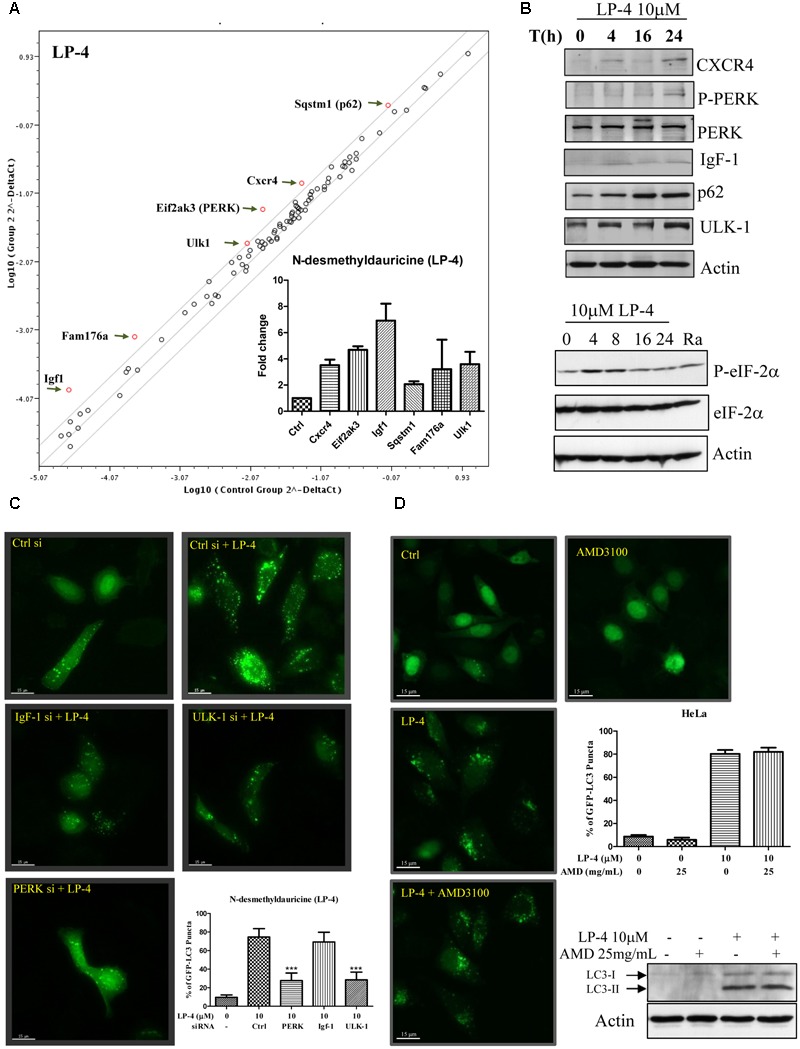
*N*-desmethyldauricine (LP-4) induces autophagy-related genes expression in HeLa cancer cells. **(A)** Autophagic gene PCR array analysis of LP-4. HeLa cells treated with LP-4 (10 μM) for 24 h were extracted for RNA before performing reverse transcription for the synthesis of cDNA. Real-time PCR reactions were performed on the 96 well plate. Scatter plot highlighted the up- or down-regulation of autophagy related genes after LP-4 treatments. **(B)** LP-4-regulated protein expression was confirmed by Western blot. Cell lysates were detected for the expression of CXCR4, P-PERK, PERK, IgF-1, p62, ULK-1 and actin, respectively. Lower panel, protein expression of P-eIF-2α, eIF-2α and actin were detected after LP-4 (10 μM) or rapamycin (Ra, 300 nM, 24 h) treatments. **(C)** Activation of PERK and ULK-1 was involved in the LP-4-regulated induction of autophagy. HeLa cells transfected with *si*RNA negative control, PERK, IgF-1 or ULK-1 *si*RNA with EGFP-LC3 plasmid for 48 h, were treated with LP-4 (10 μM) for a further 4 h before subjecting to fluorescence microscopic analysis. Bar chart represented the quantitation of autophagic cells. **(D)** CXCR4 was not required for LP-4 mediated induction of autophagy. HeLa cells transfected with EGFP-LC3 were treated with LP-4 (10 μM) in the presence of 25mg/mL CXCR4 specific inhibitor (AMD 3100) for 4 h. Bar chart indicated the autophagic activity of LP-4. The expression of LC3-II after LP-4 and AMD 3100 treatments were confirmed by Western blot. Error bars, SD ^∗∗∗^*P* < 0.001.

To study the autophagic genes that may be responsible for the induction of autophagy by LP-4, real time PCR array, which contains 87 candidate genes associated with autophagy was used. Scatter plot of genes array data showed that LP-4 up-regulated the Igf1, Fam176a, Ulk-1, PERK, Cxcr4, and Sqstm1 (p62) genes (**Figure 3A**) in HeLa cancer cells. Consistently, further validation by western blot showed that protein level of Cxcr4, p-PERK, IgF-1, Sqstm1 (p62), and Ulk-1 were increased after LP-4 treatments (**Figure 3B**) and there was an increased phosphorylation on the downstream target of PERK, the eIF2-α (Jiang et al., 2014) (**Figure 3B**, lower panel). Given the induction of autophagy can lead to the degradation of the autophagic substrate p62 (Bjorkoy et al., 2009), which is used for studying autophagic flux due to its binding ability to LC3 (Klionsky et al., 2016). In contrast, LP-4 induced the expression of p62 (**Figure 3B**). To this end, real-time PCR array was performed to analyze the transcription level of p62 mRNA after treatments of LP-4. Our results demonstrated that the increased protein level of p62 was caused by an up-regulation of the p62 mRNA level (**Figure 3A**). Therefore, results monitoring the autophagic flux by using p62 antibodies should be interpreted with cautions (Law et al., 2014).

We then validated the involvement of IgF (Dey et al., 2013), PERK (Dey et al., 2013), and Ulk-1 (Nazarko and Zhong, 2013) in LP-4-mediated autophagy through *siRNA* knockdown experiments. Knockdown of either PERK or Ulk-1 genes decreased the percentage of cells with GFP-LC3 puncta formation, whereas knockdown of IgF did not affect the percentage of cells with GFP-LC3 puncta formation significantly (**Figure 3C**). Furthermore, the induction of autophagy by LP-4 was not abolished by the addition of Cxcr4 inhibitor (AMD3100; **Figure 3D**) (Hashimoto et al., 2008). These results suggested that LP-4 may induce autophagy through the Ulk-1 and PERK dependent pathways, and did not involve Cxcr4 gene.

### LP-4 Induces Autophagy via the AMPK-mTOR Pathway

**FIGURE 4:**
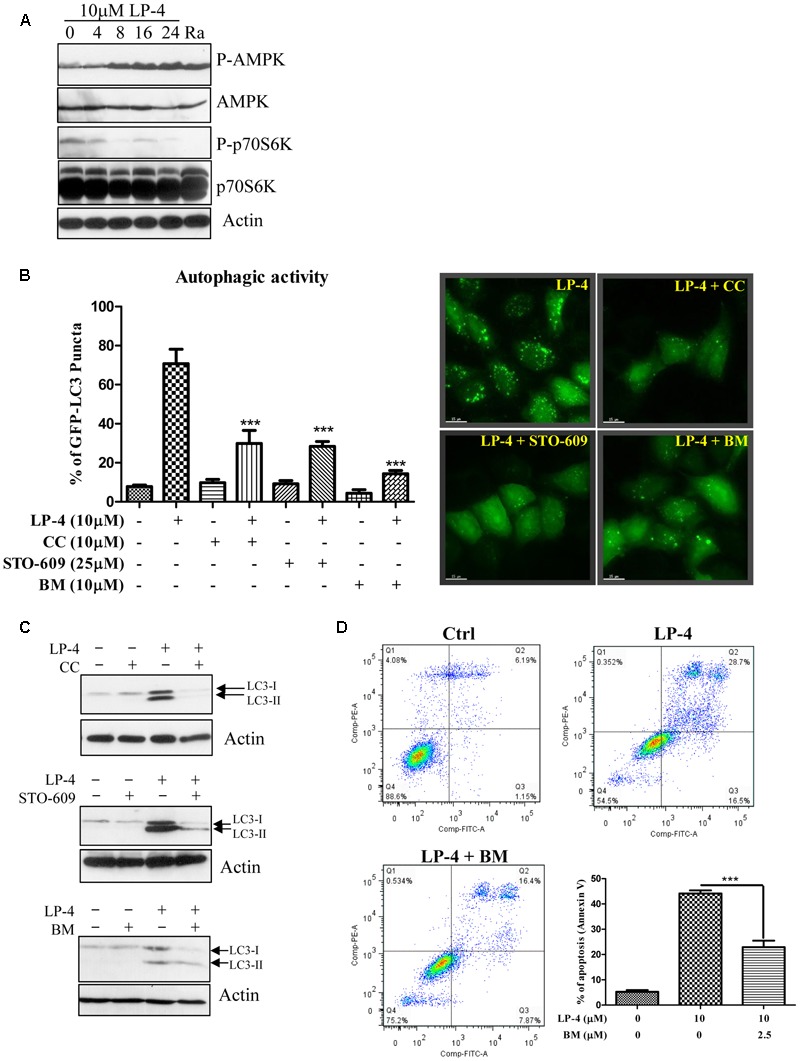
*N*-desmethyldauricine (LP-4) induces autophagy via CaMKK-β -AMPK -mTOR signaling pathways. **(A)** LP-4 activated the AMPK-mTOR cascade. Cells treated with LP-4 (10 μM) or positive control (rapamycin, 300 nM) for 24 h was analyzed for the expression of p-p70S6K, total p70S6K, p-AMPK, AMPK, and actin by Western blot. **(B)** Inhibitors for AMPK, CaMKK-β and calcium chelator inhibited autophagy induction by LP-4. EGFP-LC3 transfected HeLa cells were treated with DMSO (Ctrl) or LP-4 (10 μM) in the presence or absence of AMPK inhibitor compound C (CC, 10 μM), CaMKK β inhibitor (STO-609, 25 μM) or calcium chelator, BAPTA/AM (BM, 10 μM) for 4 h. The cells were then fixed for fluorescence microscopic analysis and quantitation of autophagic cells. **(C)** Compound C, STO-609 and BAPTA/AM inhibited the conversion of LC3-II in HeLa cells. Cells treated with LP-4 (10 μM) with or without the presence of compound C (CC, 10 μM), STO-609 (25 μM) or BAPTA/AM (10 μM) for 4 h were analyzed for the protein expression of LC3-II (LC3-I, 18 kDa; LC3-II, 16 kDa). **(D)** Calcium chelator (BAPTA/AM) suppressed the cell death induced by LP-4 in HeLa. Cells treated with LP-4 (10 μM) with the presence of BAPTA/AM (BM, 2.5 μM) for 24 h were subjected to flow cytometry analysis after annexin V staining. Bar chart indicated the percentage of apoptotic cells after treatments. Data were the means of three independent experiments; error bars, *SD* ^∗∗∗^*P* < 0.001; scale bar, 15 μm.

Extensive studies have shown that autophagy is promoted by AMPK, which is an energy sensor responsible for regulating cellular metabolism or energy homeostasis under low intracellular ATP conditions such as nutrient deprivation or hypoxia, through the AMPK-mTOR-dependent pathway (Kim et al., 2011). As shown in **Figure 4A**, there was a time-dependent increase in the phosphorylation of AMPK after treatments of LP-4. As a downstream target of mTOR, the phosphorylation of p70S6K was also decreased after LP-4 treatments (**Figure 4A**). The involvement of AMPK in LP-4-induced autophagy was further confirmed by the addition of AMPK inhibitor (compound C). As shown in **Figure 4B**, a reduction in the percentage of cells with GFP-LC3 puncta formation was observed in cells treated with both compound C and LP-4. The results suggested that LP-4 induces autophagy via the AMPK-mTOR dependent signaling cascade.

On the other hand, Ca^2+^ mobilizing agents can also lead to the activation of autophagy via the CaMKKβ-AMPK-mTOR signaling pathway (Hoyer-Hansen et al., 2007). To this end, HeLa cells were treated with LP-4 with the addition of the CaMKK-β inhibitor (STO-609) (Tokumitsu et al., 2002) or the intracellular Ca^2+^ chelator (BAPTA/AM, BM). As shown in **Figure 4B**, both STO-609 and BAPTA/AM could reduce the percentage of cells with GFP-LC3 puncta formation (**Figure 4B**), suggesting the possible role of calcium in regulating LP-4-induced autophagy. In the presence of compound C, STO-609 or BAPTA/AM, the protein level of LC3-II were reduced upon treatments of LP-4 (**Figure 4C**). Consistently, the presence of BAPTA/AM, BM resulted in significantly lower cytotoxicity in cells after LP-4 treatments (**Figure 4D**). Taken together, the results suggested the involvement of calcium in LP-4 mediated autophagy and cytotoxicity.

### LP-4 Induces Autophagy via An Increase in the Level of Cytosolic Calcium [Ca^2+^]

**FIGURE 5:**
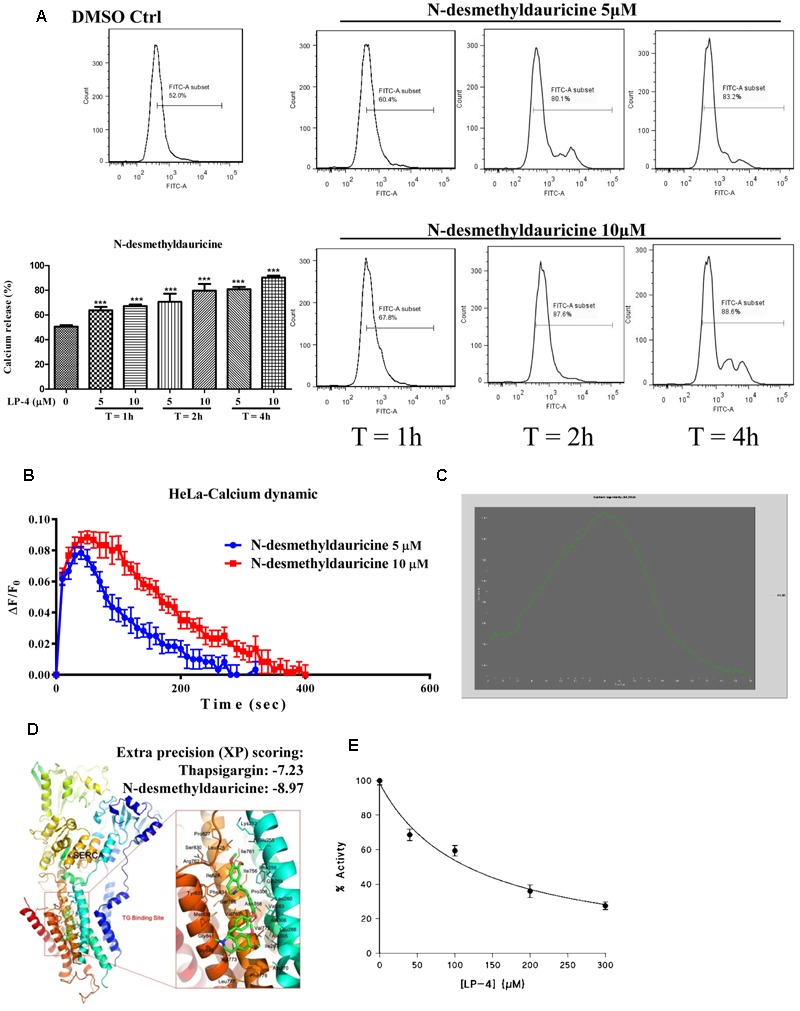
*N*-desmethyldauricine (LP-4) mobilizes cytosolic calcium in HeLa cells. **(A)** LP-4 dose- and time- dependently increased the cytosolic calcium level in HeLa cancer cells. HeLa cells treated with LP-4 (10 μM) were subjected to flow cytometry analysis after staining of Fluo-3. Representative FACS charts indicating the intracellular calcium level of cells were showed. Bar charts represented the percentage of calcium release in cells; error bars, SD ^∗∗∗^*P* < 0.001. **(B)** LP-4 induced calcium dynamic change in HeLa. HeLa cells stained with FLIPR Calcium 6 Assay Kit were treated with 5 and 10 μM of LP-4, then immediately subjected to calcium dynamic measurement by FLIPR Tetra High-Throughput Cellular Screening System. Data from the chart represents mean values ± SD of three independent experiments. **(C)** LP-4 mobilized cytosolic calcium level in HeLa cells. HeLa cells seeded in 35 mm confocal disk were incubated with 5 μM of Fluo 3/AM in HBSS buffer at 37°C for 30 min. Cells were then washed with HEPES buffer saline and incubated at 37°C for another 10 min. Calcium signal was monitored by Applied Precision DeltaVision Elite in real-time mode for consecutive 3 min during the addition of 10 μM LP-4 in HBSS buffer. Chart represents the mean intensity of fluorescence signal at 523 nm. **(D)** Computation docking of SERCA pump with LP-4. The SERCA protein was represented as carton. Key residues around the binding pocket were shown as lines and the LP-4 was presented as sticks. The hydrogen bonds were labeled as red dashed lines. Extra precision (XP) scoring for thapsigargin and LP-4 were –7.23 and –8.97, respectively. **(E)** LP-4 inhibited the activity of SERCA in skeletal muscle SR. The enzymatic assay and all measurements were performed at 25°C (pH 7.2) (Law et al., 2010).

To further confirm the role of calcium in LP-4-mediated induction of autophagy, the cytosolic [Ca^2+^] levels of HeLa cells were measured by flow cytometry. As shown in **Figure 5A**, HeLa cells incubated with LP-4 showed a dose- and time-dependent increase in fluorescence signal as revealed by the staining of Fluo 3, a highly sensitive dye for measurement of calcium in cells. Consistently, FLIPR Calcium 6 assay further demonstrated that the LP-4 dose-dependently induced calcium dynamic change in HeLa cancer cells (**Figure 5B**). Furthermore, single live-cell Ca^2+^ imaging was monitored and results showed that HeLa cells loaded with Fluo 3-AM displayed a dramatic increase in fluorescence intensity upon LP-4 (10 μM) treatment (**Figure 5C** and Supplementary Video-3). Since the inhibition of the SERCA pump can lead to the induction of autophagy through calcium homeostasis (Hoyer-Hansen et al., 2007), the molecular interactions between LP-4 and SERCA were then predicted by computational virtual ligand docking analysis. As revealed by the comparative analysis of the low-energy ligand conformations (**Figure 5D**), the preferred binding site for LP-4 is close to the binding site of a well-known inhibitor of SERCA, thapsigargin (TG), which induces autophagy through elevating intracellular calcium level in cells (Hoyer-Hansen and Jaattela, 2007). Furthermore, as shown by the predicted binding pose of LP-4 (**Figure 5D**), the hydrophobic groups bind into the hydrophobic pocket, making favorable hydrophobic effects and van der Waals interactions with residues Phe256, Leu260, Val263, Leu266, Ile267, Ala270, Ala305, Ala306, Pro308, Ile756, Ile761, Val769, Val772, Val773, Phe776, Leu777, Pro827, Leu828, Ile829, Phe834, Met838, Gly841, and Gly842, suggesting the structures of LP-4 docked into the SERCA binding site of TG. Comparison of the docking score of LP-4 (-8.97 kcal/mol) with TG (-7.23 kcal/mol) indicated that both compounds were located within the SERCA binding pocket.

To further validate the computational docking results, the effect of LP-4 on the activity of SERCA1A isoform was evaluated by using purified rabbit skeletal muscle sarcoplasmic reticulum (SR) membranes (Wu et al., 1995). As shown in **Figure 5E**, although SERCA activity was inhibited by LP-4 in a dose-dependent manner, the IC_50_ value of LP-4 was very high, appropriately 100 μM, which suggested that LP-4 is a weak SERCA inhibitor.

### LP-4 Induces Autophagic Cell Death in Apoptosis-Resistant Cancer Cells

**FIGURE 6:**
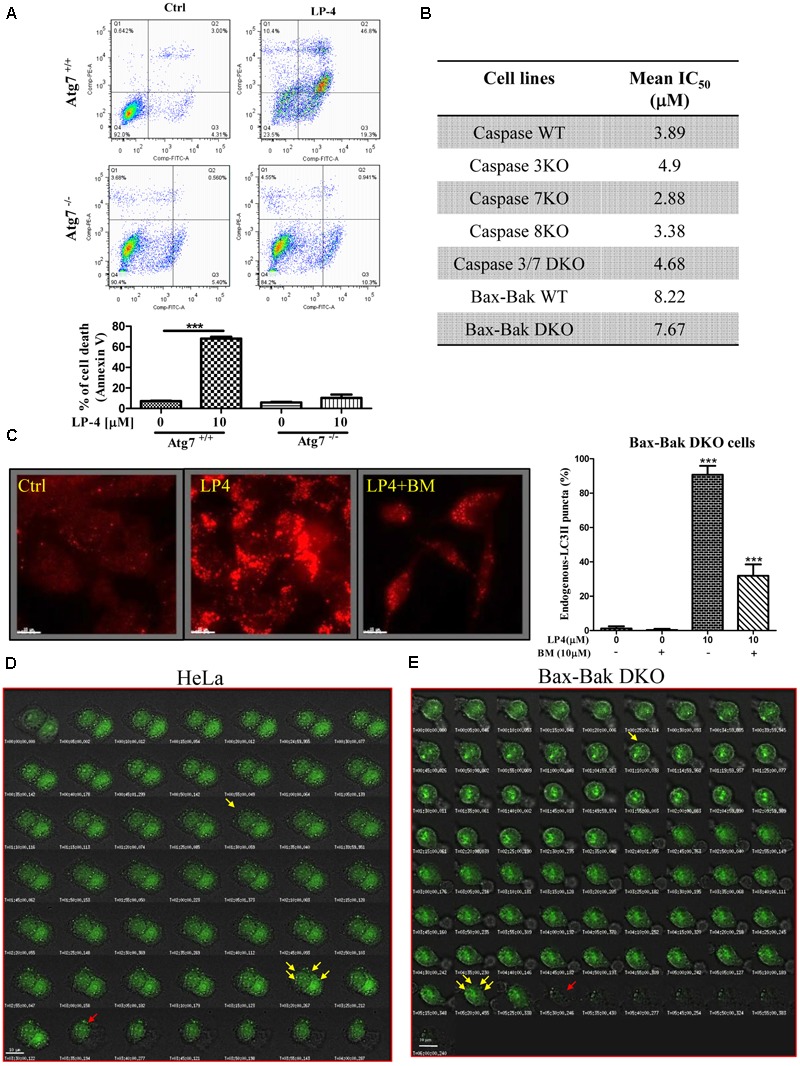
*N*-desmethyldauricine (LP-4) induces autophagic cell death in apoptosis resistant cells. **(A)** Cell death measurement (%) in Atg7- wild-type and -deficient MEFs after LP-4 treatment. **(B)** Cytotoxicity (IC_50_ value) of LP-4 in caspase WT/-3/-7/-8, caspase -3 and -7 DKO, Bax-Bak DKO -wild-type and -deficient MEFs. These panel of MEFs were incubated with LP-4 (0.19–100 μM) for 3 days. MTT assay were performed to evaluate the mean IC_50_ of LP-4. **(C)** BAPTA/AM inhibited the autophagic effect of LP-4 in apoptosis-resistant cells. Bax-Bak DKO deficient MEFs were treated with 10 μM of LP-4 in the presence of calcium chelator, BAPTA/AM (BM, 10 μM), for 4 h. The cells were then fixed and incubated with anti-LC3B and TRITC-conjugated secondary antibodies before fluorescence microscopic analysis. **(D,E)** Live-cell imaging of LP-4-induced autophagic cell death. Cells transfected with EGFP-LC3 were treated with 10 μM of LP-4. Cells were then placed inside the 37°C imaging chamber supplied with 5% of CO_2_ for monitoring of autophagy and autophagic cell death. Live-cell fluorescent and bright field images were captured for 4 h with 10 min time-intervals. Yellow arrows indicated autophagy induction with EGFP-LC3 accumulation; Red arrows indicated autophagic cell death. ^∗∗∗^*P* < 0.001.

Although autophagy can act as a tumor-suppression mechanism, it is also a mechanism for stress tolerance which may sustain cancer cells viability under adverse conditions and promote pathogenesis of cancers (White and DiPaola, 2009). To investigate the LP-4-mediated cell death mechanism, both Atg7 wild-type and deficient MEFs treated with LP-4 were subjected to annexin V flow cytometry analysis. As shown in **Figure 6A**, LP-4 possessed significantly higher cytotoxicity in Atg7 wild-type MEFs (c.a. 70% of cell death upon 10 μM LP-4 treatment), when compared to Atg7 deficient MEFs (c.a. 10% cell death upon 10 μM LP-4 treatment). As the failure of the induction of autophagy in Atg7^-/-^ deficient MEFs could lead to a significant decrease in the percentage of cell death, therefore, it was suggested that LP-4 may lead to autophagic cell death. We further investigated the cytotoxic potency of LP-4 toward a panel of apoptosis -resistant or -defective cells, including caspase -3/-7/-8/ deficient MEFs, caspase-3/-7 double knockout (DKO) MEFs and Bax-Bak DKO MEFs. Our results demonstrated that LP-4 displayed no significant difference in the cytotoxicity effect (IC_50_ value) toward the panel of selected apoptosis -resistant or -defective cells (**Figure 6B**). The data supported our postulation that LP-4 could induce autophagic cell death that is independent of the apoptosis pathway.

In fact, the use of autophagy inducers to induce autophagic cell death in apoptosis -resistant cells has been considered as an effective alternative approaches in treating cancers (Alva et al., 2004). To confirm whether LP-4 could induce autophagy via the mobilization of calcium in apoptosis-resistant cells, Bax-Bak DKO MEFs treated with LP-4 were incubated with calcium chelator (BAPTA/AM) and then subjected to immunofluorescence staining. As demonstrated by a significant decrease of fluorescent endogenous LC3B signal (**Figure 6C**), the chelation of calcium by BAPTA/AM abolished LP-4 induced autophagy. This result suggested that LP-4 is able to mobilize calcium release, which induces autophagy in apoptosis-resistant cells. Furthermore, the induction of autophagy and autophagic cell death by LP-4 in HeLa and Bax-Bak DKO apoptosis-resistant MEFs were monitored by live cell imaging (**Figures 6D,E** and Supplementary Videos-1, 2).

**FIGURE 7:**
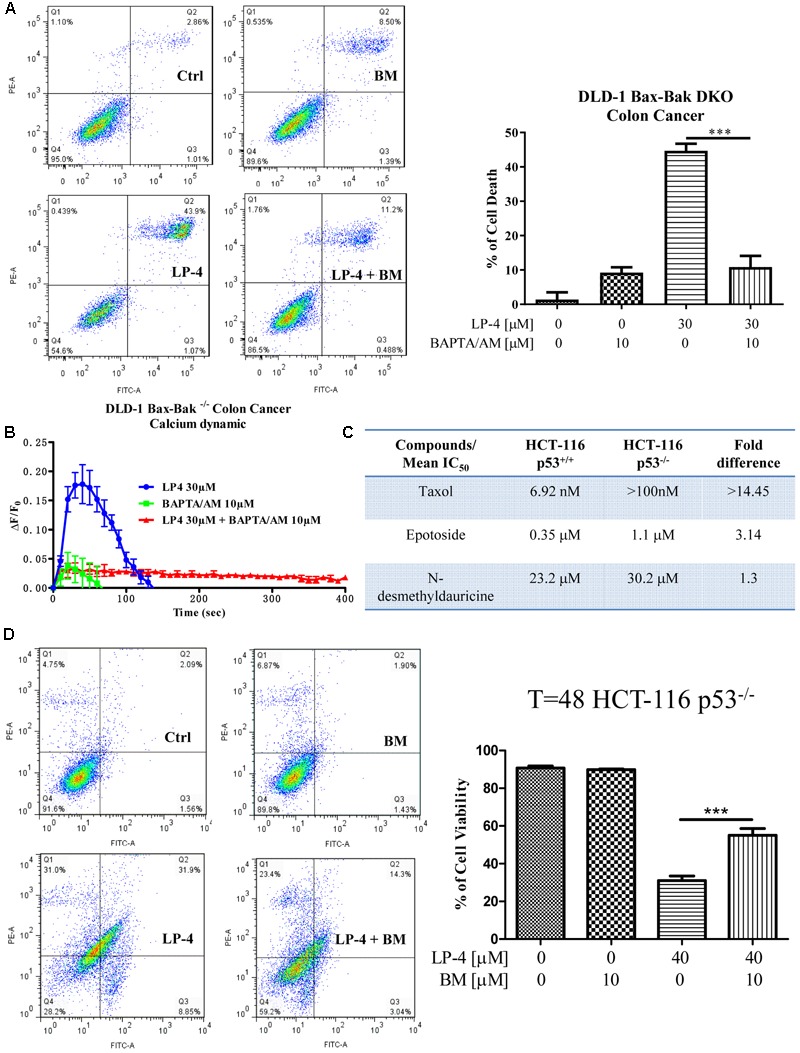
*N*-desmethyldauricine (LP-4) induces autophagic cell death in apoptosis resistant cancer cells. **(A)** BAPTA/AM inhibited cell death induced by LP-4 in apoptosis-resistant cancer cells. DLD-1 Bax-Bak DKO colon cancer cells treated with LP-4 (30 μM) in the presence of 10 μM of BAPTA/AM for 24 h were assayed by flow cytometry after annexin V staining. **(B)** BAPTA/AM abolished the LP-4-induced calcium dynamic change in apoptosis-resistant cancer cells. DLD-1 Bax-Bak DKO colon cancer cells stained with FLIPR Calcium 6 Assay Kit were treated with 30 μM of LP-4, with or without pretreatment of BAPTA/AM (10 μM), then immediately subjected to calcium dynamic measurement by FLIPR Tetra High-Throughput Cellular Screening System. Data from the chart represents mean values ± SD of three independent experiments. **(C)** Drug-resistant effects of LP-4 on p53-deficient apoptosis-resistant cancer cells. HCT-116 p53 wild-type and deficient colon cancer cells were incubated with different concentrations of LP-4, taxol and etoposide for 3 days. Cytotoxicity of these compounds were measured by MTT assay and shown as the mean IC_50_. **(D)** BAPTA/AM recovered the cell death from LP-4 treatment in p53-deficient apoptosis-resistant cancer cells. HCT-116 p53-deficient colon cancer cells treated with LP-4 (10 μM) in the presence of 10 μM of BAPTA/AM for 24 h were assayed by flow cytometry after annexin V staining. ^∗∗∗^*P* < 0.001.

To investigate the role of autophagy in LP-4-mediated cell death in cancer, DLD-1 Bax-Bak DKO apoptosis-resistant colon cancer cells treated with LP-4 and calcium chelator (BAPTA/AM) were subjected to annexin V flow analysis. While LP-4 markedly induced autophagic cell death in Bax-Bak DKO apoptosis-resistant cancer cells, BAPTA/AM significantly suppressed the LP4-induced cell death (**Figure 7A**). Concomitantly, LP-4 alone markedly stimulated the calcium dynamic change in DLD-1 Bax-Bak DKO cancer cells, whereas addition of calcium chelator, BAPTA/AM completely suppressed the LP-4-mediated calcium flux in these apoptosis-resistant cancer cells (**Figure 7B**). Collectively, these data suggested that LP-4 could induce cell death through autophagy in apoptosis-resistant cancer cells via calcium mobilization. In p53 knockout apoptosis-resistant cancer cells, taxol and etoposide known for inducing cancer cell death via the p53 pathway independent of autophagy (Xie et al., 2011; Peng et al., 2014), the differences in their mean IC_50_ value between the p53-deficient and wild-type HCT-116 cells were significantly higher (14.45- and 3.14-fold) (**Figure 7C**). In contract, LP-4 showed a mean IC_50_ value of 23.2 μM in wild-type HCT 116 p53^+/+^ compared to 30.2 μM in HCT 116 p53^-/-^ cells, respectively, demonstrating merely 1.3-fold differences in cytotoxicity (**Figure 7C**). Concomitantly, addition of BAPTA/AM markedly recovered the cell death from LP-4 treatment in p53-deficient HCT-116 cancer cells (**Figure 7D**). These results suggested that LP-4-mobilized calcium release may circumvent the apoptosis-resistant phenotype of cancer cells caused by genetic deficiency in p53 gene.

## Discussion

Autophagy plays an essential role in defense against cancers, neurodegenerative disorders, aging, and infectious diseases (Mizushima et al., 2008). Small-molecules that induce autophagy may have broad therapeutics applications. In our previous studies, we have identified several triterpenoid and alkaloid compounds as autophagic enhancers targeting apoptosis-resistant cells which can potentially be developed into new anti-cancer agents (Law et al., 2010; Wong et al., 2013; Law et al., 2014). Among these compounds, the triterpenoid saikosaponin-d (Ssd) and alisol B were found to directly inhibit SERCA, leading to release of cytosolic [Ca^2+^], and thereby induced autophagy and significant autophagic cytotoxicity upon various cancer cells. The natural alkaloid small-molecules, including liensinine, isoliensinine, dauricine, and cepharanthine, also stimulate the AMPK-mTOR dependent induction of autophagy and autophagic cell death in a panel of apoptosis-resistant cells. However, the role of cytosolic calcium level in such alkaloid-induced autophagic cytotoxicity is unclear and the resulted cell death-inducing effect appeared to be mild. In this study, we used a derivative of dauricine (*N*-desmethyldauricine/LP-4) to further verify the precise cellular machinery regulating the autophagic cell death. Derivatives of dauricine are of interest since dauricine has been shown *in vitro* to process anti-cancer property in colon (Yang et al., 2010), lung (Jin et al., 2010), breast (Tang et al., 2009), and urinary cancers (Wang et al., 2012). In fact, our previous findings point toward potential use of dauricine in multidrug- and apoptosis-resistant cancers intervention (Law et al., 2014). Intriguingly, LP-4 demonstrated a better therapeutic effect than dauricine upon some experimental cancer models. For example, we previously found that, dauricine induced autophagic cell death with a higher IC_50_ value (Law et al., 2014) toward the cell lines MCF-7 (28.7 μM) and A549 (40.4 μM), while the IC_50_ value corresponding to these cell lines upon LP-4 treatment are 1.8 to 2-fold lower as demonstrated in this report: MCF-7 (15.5 μM) and A549 (19.7 μM). Such higher cytotoxicity could be a result of the existence of the electrophilic quinone methide group on LP-4 (Wang et al., 2009; Jin et al., 2010). However, the molecular mechanisms for inducing the higher cellular toxicity as demonstrated might be attributed to increased autophagic effect (unpublished observation).

Thus far, information concerning the detail mechanisms underpinning LP-4 function is scarced, our pilot study here correlated LP-4 and the induction of autophagy via the manipulation of cytosolic Ca^2+^ concentration. We firstly demonstrated that LP-4 interacts with SERCA inhibiting the transfer of Ca^2+^ from cytoplasm to the ER lumen resulted in the accumulation of cytosolic Ca^2+^. Result collected from the FLIPR assay (**Figure 7B**) confirmed the capability of LP-4 in triggering Ca^2+^ release, since pretreatment of the calcium chelator BATA/AM abolished the calcium dynamics of our LP-4-treated Bax-Bak double knockout DLD-1 colon cancer cells. The increase in cytosolic level of Ca^2+^ further activates the calcium dependent kinase, CaMKK-β, for the activation of AMPK-mTOR signaling cascade (**Figure 4**) and subsequently induces autophagy as well as autophagic cell death in cancer cells. The cytotoxic effect of LP-4 significantly reduced when the apoptosis-resistant Bax-Bak double knockout DLD-1 colon cancer cells were receiving the same treatment (**Figure 7A**) consolidated that the LP-4-induced cytotoxicity is related to calcium-mediated autophagic cell death.

In contrast to the prominent Ca^2+^ mobilizing ability, the interaction between LP-4 and SERCA is comparatively weak (50% reduction of SERCA activity in response to 150 μM LP-4 treatment). Such observation implied that, LP-4 may also intervene with other calcium transporters which influence the localization of cytosolic Ca^2+^. Other possible coupling partners of LP-4 include the 1,4,5-trisphosphate receptor (InsP3R) and the RyRs localized in the SR/ER (Otsu et al., 1990; Nixon et al., 1994). The InsP3R is membrane glycoprotein complex which is an important Ca^2+^ channel responsible for the release of Ca^2+^ from intracellular pool upon inositol trisphosphate (InsP3) activation (Marchant and Taylor, 1997). InsP3R is critical to the regulation of various cellular processes, such as cell division, proliferation, apoptosis, and etc. (Bosanac et al., 2002). Of note, InsP3R-mediated release of Ca^2+^ from the ER has been reported during cellular starvation which leads to the upregulation of autophagy as a result of the elevated cytosolic Ca^2+^ level (Decuypere et al., 2011). Similar to the InsP3R, the RyRs constituting a family of Ca^2+^ release channels and mediate the release of calcium ions from the SR/ER. The RyRs channels are ubiquitously expressed in many types of cells and participate in a variety of vital Ca^2+^ signaling for maintaining cellular homeostasis (Fill and Copello, 2002). Bround et al. (2013) demonstrated that one of the isoforms of RyRs, the RyR2, can act as proximal controllers of mitochondrial Ca^2+^, ATP levels, and a cascade of transcription factors controlling metabolism and survival via the regulation of Ca^2+^ fluxes. In addition, the loss of RyR2 receptor could induce a non-apoptotic form of programmed cell death associated with increased calpain-10 but not caspase-3 activation or ER stress (Bround et al., 2013). Another recent study also suggested the RyRs type 3 (RyR3) can trigger autophagic cell death in hippocampal neural stem cells via its regulatory function of ER Ca^2+^ mobilization (Chung et al., 2016). Together with our findings, targeting the Ca^2+^ signaling pathway by disrupting the cytosolic Ca^2+^ level to induce autophagic cell death in apoptosis- and drug-resistant cancers appeared to be a promising therapeutic strategy. Since, the normal molecular regulation of Ca^2+^ channels during tumorigenesis are generally hampered, cancer cells are more vulnerable to treatment associated with the alternation of Ca^2+^ mobilization owing to the loss of redundancy in Ca^2+^ channels (Ding et al., 2010). In line with this idea, we have showed that the use of LP-4 specifically induced cytotoxicity toward different cancer cells instead of the normal cellular counterpart (**Figure 1B**). It is worth notice that, such cancer-targeting cytotoxic effect of LP-4 may also be associated with the cell type-specific nature of autophagic functions. The molecular machineries operating in different cell types are varied, their crosstalk with the complex autophagy signaling pathways lead to the discrepancy in autophagy-induced cellular functions. Since, the molecular network constituting cancer cells and normal cells are different which may account for the specificity of the LP-4 drug action as illustrated in our data.

In summary, LP-4 induces cytotoxicity bypasses the apoptotic machinery and is associated with autophagy induction which leads to autophagic cell death. The LP-4-induced autophagy is mediated by the alteration of cytosolic Ca^2+^ level via the manipulation of cellular Ca^2+^ transportation system. Therefore, compounds which are capable of interfering with Ca^2+^ signaling are having the therapeutic potential for clinical application against cancers with phenotypes resistant to apoptosis and conventional chemotherapy. Findings acquired from this report also provided insight into and suggested an effective experimental platform for the search of other Ca^2+^ signaling modulators for refractory cancer therapy through autophagy upregulation.

## Author Contributions

BL, SM, JC, and VW designed, carried out the experiments, analyzed the data and prepared the draft of manuscript. Z-HJ provided the compounds for experiment. FM and M-HJ conducted the SERCA activity assay. W-WX and X-JY performed the computational docking. S-WX and JG participated the experiments. PC prepared the chemical structure. LL and VW conceived the idea, supervised all research and revised the manuscript. All authors reviewed the manuscript.

## Conflict of Interest Statement

The authors declare that the research was conducted in the absence of any commercial or financial relationships that could be construed as a potential conflict of interest.

## Footnotes

1 http://pubchem.ncbi.nlm.nih.gov

## Abbreviations

AMPK: AMP-activated protein kinase
Atg: autophagy-related gene
CaMKKβ: Ca^2+^/calmodulin-dependent kinase kinase-β
ER: endoplasmic reticulum
InsP_3_: inositol 1,4,5-trisphosphate
LC3: light-chain 3
LP-4: *N*-desmethyldauricine
MEFs: mouse embryonic fibroblasts
PERK: pancreatic ER kinase
Ryr: ryanodine receptor
SERCA: sarcoplasmic/endoplasmic reticulum Ca^2+^ ATPase pump
UPR: unfolded protein responses
3-MA: 3-methyladenine

## References

[B1] Alva A. S. Gultekin S. H. Baehrecke E. H. (2004). Autophagy in human tumors: Cell survival or death? *Cell Death Differ.* 11 1046–1048. 10.1038/sj.cdd.4401445 15143348

[B2] Berridge M. J. Lipp P. Bootman M. D. (2000). The versatility and universality of calcium signalling. *Nat. Rev. Mol. Cell Biol.* 1 11–21. 10.1038/35036035 11413485

[B3] Bjorkoy G. Lamark T. Pankiv S. Overvatn A. Brech A. Johansen T. (2009). Monitoring autophagic degradation of p62/SQSTM1. *Methods Enzymol.* 452 181–197. 10.1016/s0076-6879(08)03612-4 19200883

[B4] Bosanac I. Alattia J. R. Mal T. K. Chan J. Talarico S. Tong F. K. (2002). Structure of the inositol 1,4,5-trisphosphate receptor binding core in complex with its ligand. *Nature* 420 696–700. 10.1038/nature01268 12442173

[B5] Bround M. J. Wambolt R. Luciani D. S. Kulpa J. E. Rodrigues B. Brownsey R. W. (2013). Cardiomyocyte ATP production, metabolic flexibility, and survival require calcium flux through cardiac ryanodine receptors in vivo. *J. Biol. Chem.* 288 18975–18986. 10.1074/jbc.M112.427062 23678000 PMC3696672

[B6] Bursch W. Ellinger A. Kienzl H. Török L. Pandey S. Sikorska M. (1996). Active cell death induced by the anti-estrogens tamoxifen and ICI 164 384 in human mammary carcinoma cells (MCF-7) in culture: the role of autophagy. *Carcinogenesis* 17 1595–1607. 10.1093/carcin/17.8.1595 8761415

[B7] Chang C. P. Yang M. C. Liu H. S. Lin Y. S. Lei H. Y. (2007). Concanavalin A induces autophagy in hepatoma cells and has a therapeutic effect in a murine in situ hepatoma model. *Hepatology* 45 286–296. 10.1002/hep.21509 17256764

[B8] Chung K. M. Jeong E. J. Park H. An H. K. Yu S. W. (2016). Mediation of autophagic cell death by type 3 ryanodine receptor (RyR3) in adult hippocampal neural stem cells. *Front. Cell Neurosci.* 10:116 10.3389/fncel.2016.00116 PMC4858590 27199668

[B9] Dalby K. N. Tekedereli I. Lopez-Berestein G. Ozpolat B. (2010). Targeting the prodeath and prosurvival functions of autophagy as novel therapeutic strategies in cancer. *Autophagy* 6 322–329. 10.4161/auto.6.3.11625 20224296 PMC2914492

[B10] Decuypere J. P. Welkenhuyzen K. Luyten T. Ponsaerts R. Dewaele M. Molgo J. (2011). Ins(1,4,5)P3 receptor-mediated Ca^2+^ signaling and autophagy induction are interrelated. *Autophagy* 7 1472–1489. 10.4161/auto.7.12.17909 22082873 PMC3327615

[B11] Dey S. Tameire F. Koumenis C. (2013). PERK-ing up autophagy during MYC-induced tumorigenesis. *Autophagy* 9 612–614. 10.4161/auto.23486 23328692 PMC3627677

[B12] Ding X. He Z. Zhou K. Cheng J. Yao H. Lu D. (2010). Essential role of TRPC6 channels in G2/M phase transition and development of human glioma. *J. Natl. Cancer Inst.* 102 1052–1068. 10.1093/jnci/djq217 20554944

[B13] Fill M. Copello J. A. (2002). Ryanodine receptor calcium release channels. *Physiol. Rev.* 82 893–922. 10.1152/physrev.00013.2002 12270947

[B14] Hashimoto I. Koizumi K. Tatematsu M. Minami T. Cho S. Takeno N. (2008). Blocking on the CXCR4/mTOR signalling pathway induces the anti-metastatic properties and autophagic cell death in peritoneal disseminated gastric cancer cells. *Eur. J. Cancer* 44 1022–1029. 10.1016/j.ejca.2008.02.043 18375114

[B15] Hasima N. Ozpolat B. (2014). Regulation of autophagy by polyphenolic compounds as a potential therapeutic strategy for cancer. *Cell Death Dis.* 5:e1509 10.1038/cddis.2014.467 PMC4260725 25375374

[B16] Hoyer-Hansen M. Bastholm L. Mathiasen I. S. Elling F. Jaattela M. (2005). Vitamin D analog EB1089 triggers dramatic lysosomal changes and Beclin 1-mediated autophagic cell death. *Cell Death Differ.* 12 1297–1309. 10.1038/sj.cdd.4401651 15905882

[B17] Hoyer-Hansen M. Bastholm L. Szyniarowski P. Campanella M. Szabadkai G. Farkas T. (2007). Control of macroautophagy by calcium, calmodulin-dependent kinase kinase-βeta, and Bcl-2. *Mol. Cell* 25 193–205. 10.1016/j.molcel.2006.12.009 17244528

[B18] Hoyer-Hansen M. Jaattela M. (2007). Connecting endoplasmic reticulum stress to autophagy by unfolded protein response and calcium. *Cell Death Differ.* 14 1576–1582. 10.1038/sj.cdd.4402200 17612585

[B19] Jiang P. Mizushima N. (2014). Autophagy and human diseases. *Cell Res.* 24 69–79. 10.1038/cr.2013.161 24323045 PMC3879707

[B20] Jiang Q. Li F. Shi K. Wu P. An J. Yang Y. (2014). Involvement of p38 in signal switching from autophagy to apoptosis via the PERK/eIF2alpha/ATF4 axis in selenite-treated NB4 cells. *Cell Death Dis.* 5:e1270 10.1038/cddis.2014.200 PMC4047911 24874742

[B21] Jin H. Dai J. Chen X. Liu J. Zhong D. Gu Y. (2010). Pulmonary toxicity and metabolic activation of dauricine in CD-1 mice. *J. Pharmacol. Exp. Ther.* 332 738–746. 10.1124/jpet.109.162297 20008063

[B22] Juenemann K. Reits E. A. (2012). Alternative macroautophagic pathways. *Int. J. Cell Biol.* 2012:189794 10.1155/2012/189794 PMC3320029 22536246

[B23] Kim J. Kundu M. Viollet B. Guan K. L. (2011). AMPK and mTOR regulate autophagy through direct phosphorylation of Ulk1. *Nat. Cell Biol.* 13 132–141. 10.1038/ncb2152 21258367 PMC3987946

[B24] Klionsky D. J. Abdelmohsen K. Abe A. Abedin M. J. Abeliovich H. Acevedo Arozena A. (2016). Guidelines for the use and interpretation of assays for monitoring autophagy (3rd edition). *Autophagy* 12 1–222. 10.1080/15548627.2015.1100356 26799652 PMC4835977

[B25] Kondo Y. Kanzawa T. Sawaya R. Kondo S. (2005). The role of autophagy in cancer development and response to therapy. *Nat. Rev. Cancer* 5 726–734. 10.1038/nrc1692 16148885

[B26] Kuma A. Matsui M. Mizushima N. (2007). LC3, an autophagosome marker, can be incorporated into protein aggregates independent of autophagy: caution in the interpretation of LC3 localization. *Autophagy* 3 323–328. 10.4161/auto.4012 17387262

[B27] Law B. Y. Chan W. K. Xu S. W. Wang J. R. Bai L. P. Liu L. (2014). Natural small-molecule enhancers of autophagy induce autophagic cell death in apoptosis-defective cells. *Sci. Rep.* 4:5510 10.1038/srep05510 PMC4076737 24981420

[B28] Law B. Y. Wang M. Ma D. L. Al-Mousa F. Michelangeli F. Cheng S. H. (2010). Alisol B, a novel inhibitor of the sarcoplasmic/endoplasmic reticulum Ca^2+^ ATPase pump, induces autophagy, endoplasmic reticulum stress, and apoptosis. *Mol. Cancer Ther.* 9 718–730. 10.1158/1535-7163.mct-09-0700 20197400

[B29] Levine B. Kroemer G. (2008). Autophagy in the pathogenesis of disease. *Cell* 132 27–42. 10.1016/j.cell.2007.12.018 18191218 PMC2696814

[B30] Maiuri M. C. Zalckvar E. Kimchi A. Kroemer G. (2007). Self-eating and self-killing: crosstalk between autophagy and apoptosis. *Nat. Rev. Mol. Cell Biol.* 8 741–752. 10.1038/nrm2239 17717517

[B31] Marchant J. S. Taylor C. W. (1997). Cooperative activation of IP3 receptors by sequential binding of IP3 and Ca^2+^ safeguards against spontaneous activity. *Curr. Biol.* 7 510–518. 10.1016/S0960-9822(06)00222-3 9210378

[B32] Michelangeli F. Colyer J. East J. M. Lee A. G. (1990). Effect of pH on the activity of the Ca^2+^ + Mg^2+^-activated ATPase of sarcoplasmic reticulum. *Biochem. J.* 267 423–429. 10.1042/bj2670423 2139777 PMC1131306

[B33] Michelangeli F. Munkonge F. M. (1991). Methods of reconstitution of the purified sarcoplasmic reticulum (Ca(2+)-Mg2+)-ATPase using bile salt detergents to form membranes of defined lipid to protein ratios or sealed vesicles. *Anal. Biochem.* 194 231–236. 10.1016/0003-2697(91)90223-G 1830725

[B34] Mizushima N. Levine B. Cuervo A. M. Klionsky D. J. (2008). Autophagy fights disease through cellular self-digestion. *Nature* 451 1069–1075. 10.1038/nature06639 18305538 PMC2670399

[B35] Mizushima N. Yoshimori T. (2007). How to interpret LC3 immunoblotting. *Autophagy* 3 542–545. 10.4161/auto.4600 17611390

[B36] Monteith G. R. Davis F. M. Roberts-Thomson S. J. (2012). Calcium channels and pumps in cancer: changes and consequences. *J. Biol. Chem.* 287 31666–31673. 10.1074/jbc.R112.343061 22822055 PMC3442501

[B37] Monteith G. R. McAndrew D. Faddy H. M. Roberts-Thomson S. J. (2007). Calcium and cancer: targeting Ca^2+^ transport. *Nat. Rev. Cancer* 7 519–530. 10.1038/nrc2171 17585332

[B38] Nazarko V. Y. Zhong Q. (2013). ULK1 targets Beclin-1 in autophagy. *Nat. Cell Biol.* 15 727–728. 10.1038/ncb2797 23817237 PMC4442023

[B39] Nixon G. F. Mignery G. A. Somlyo A. V. (1994). Immunogold localization of inositol 1,4,5-trisphosphate receptors and characterization of ultrastructural features of the sarcoplasmic reticulum in phasic and tonic smooth muscle. *J. Muscle Res. Cell Motil.* 15 682–700. 10.1007/BF00121075 7706424

[B40] Obara K. Miyashita N. Xu C. Toyoshima I. Sugita Y. Inesi G. (2005). Structural role of countertransport revealed in Ca^2+^ pump crystal structure in the absence of Ca^2+^. *Proc. Natl. Acad. Sci. U.S.A.* 102 14489–14496. 10.1073/pnas.0506222102 16150713 PMC1253571

[B41] Ohsumi Y. Mizushima N. (2004). Two ubiquitin-like conjugation systems essential for autophagy. *Semin. Cell Dev. Biol.* 15 231–236. 10.1016/j.semcdb.2003.12.004 15209383

[B42] Opipari A. W. Jr. Tan L. Boitano A. E. Sorenson D. R. Aurora A. Liu J. R. (2004). Resveratrol-induced autophagocytosis in ovarian cancer cells. *Cancer Res.* 64 696–703. 10.1158/0008-5472.CAN-03-2404 14744787

[B43] Otsu K. Willard H. F. Khanna V. K. Zorzato F. Green N. M. MacLennan D. H. (1990). Molecular cloning of cDNA encoding the Ca^2+^ release channel (ryanodine receptor) of rabbit cardiac muscle sarcoplasmic reticulum. *J. Biol. Chem.* 265 13472–13483. 2380170

[B44] Pallet N. Legendre C. (2013). Adverse events associated with mTOR inhibitors. *Expert Opin. Drug Saf.* 12 177–186. 10.1517/14740338.2013.752814 23252795

[B45] Pan X. P. (1992). A new alkaloid from *Menispermum dauricum* DC–N-desmethyldauricine. *Yao Xue Xue Bao* 27 788–791. 1293929

[B46] Peng X. Gong F. Chen Y. Jiang Y. Liu J. Yu M. (2014). Autophagy promotes paclitaxel resistance of cervical cancer cells: involvement of Warburg effect activated hypoxia-induced factor 1-alpha-mediated signaling. *Cell Death Dis.* 5:e1367 10.1038/cddis.2014.297 PMC4454295 25118927

[B47] Pereira G. J. Hirata H. Fimia G. M. do Carmo L. G. Bincoletto C. Han S. W. (2011). Nicotinic acid adenine dinucleotide phosphate (NAADP) regulates autophagy in cultured astrocytes. *J. Biol. Chem.* 286 27875–27881. 10.1074/jbc.C110.216580 21610076 PMC3151033

[B48] Rashid H. O. Yadav R. K. Kim H. R. Chae H. J. (2015). ER stress: autophagy induction, inhibition and selection. *Autophagy* 11 1956–1977. 10.1080/15548627.2015.1091141 26389781 PMC4824587

[B49] Tang X. D. Zhou X. Zhou K. Y. (2009). Dauricine inhibits insulin-like growth factor-I-induced hypoxia inducible factor 1alpha protein accumulation and vascular endothelial growth factor expression in human breast cancer cells. *Acta Pharmacol. Sin.* 30 605–616. 10.1038/aps.2009.8 19349962 PMC4002832

[B50] Tanida I. Ueno T. Kominami E. (2008). LC3 and Autophagy. *Methods Mol. Biol.* 445 77–88. 10.1007/978-1-59745-157-4_4 18425443

[B51] Tokumitsu H. Inuzuka H. Ishikawa Y. Ikeda M. Saji I. Kobayashi R. (2002). STO-609, a specific inhibitor of the Ca^2+^/calmodulin-dependent protein kinase kinase. *J. Biol. Chem.* 277 15813–15818. 10.1074/jbc.M201075200 11867640

[B52] Tsujimoto Y. Shimizu S. (2005). Another way to die: autophagic programmed cell death. *Cell Death Differ.* 12(Suppl. 2) 1528–1534. 10.1038/sj.cdd.4401777 16247500

[B53] Turcotte S. Chan D. A. Sutphin P. D. Hay M. P. Denny W. A. Giaccia A. J. (2008). A molecule targeting VHL-deficient renal cell carcinoma that induces autophagy. *Cancer Cell* 14 90–102. 10.1016/j.ccr.2008.06.004 18598947 PMC2819422

[B54] Turcotte S. Giaccia A. J. (2010). Targeting cancer cells through autophagy for anticancer therapy. *Curr. Opin. Cell Biol.* 22 246–251. 10.1016/j.ceb.2009.12.007 20056398 PMC4012537

[B55] Wang J. Li Y. Zu X. B. Chen M. F. Qi L. (2012). Dauricine can inhibit the activity of proliferation of urinary tract tumor cells. *Asian Pac. J. Trop. Med.* 5 973–976. 10.1016/s1995-7645(12)60185-0 23199717

[B56] Wang Y. Zhong D. Chen X. Zheng J. (2009). Identification of quinone methide metabolites of dauricine in human liver microsomes and in rat bile. *Chem. Res. Toxicol.* 22 824–834. 10.1021/tx800397e 19358519

[B57] White E. DiPaola R. S. (2009). The double-edged sword of autophagy modulation in cancer. *Clin. Cancer Res.* 15 5308–5316. 10.1158/1078-0432.ccr-07-5023 19706824 PMC2737083

[B58] Wong V. K. Li T. Law B. Y. Ma E. D. Yip N. C. Michelangeli F. (2013). Saikosaponin-d, a novel SERCA inhibitor, induces autophagic cell death in apoptosis-defective cells. *Cell Death Dis.* 4:e720 10.1038/cddis.2013.217 PMC3730398 23846222

[B59] Wu K. D. Lee W. S. Wey J. Bungard D. Lytton J. (1995). Localization and quantification of endoplasmic reticulum Ca^2+^-ATPase isoform transcripts. *Am. J. Physiol.* 269(3 Pt 1) C775–C784. 7573409 10.1152/ajpcell.1995.269.3.C775

[B60] Wu M. Lao Y. Xu N. Wang X. Tan H. Fu W. (2015). Guttiferone K induces autophagy and sensitizes cancer cells to nutrient stress-induced cell death. *Phytomedicine* 22 902–910. 10.1016/j.phymed.2015.06.008 26321739

[B61] Xie B. S. Zhao H. C. Yao S. K. Zhuo D. X. Jin B. Lv D. C. (2011). Autophagy inhibition enhances etoposide-induced cell death in human hepatoma G2 cells. *Int. J. Mol. Med.* 27 599–606. 10.3892/ijmm.2011.607 21274505

[B62] Yang Z. Li C. Wang X. Zhai C. Yi Z. Wang L. (2010). Dauricine induces apoptosis, inhibits proliferation and invasion through inhibiting NF-kappaB signaling pathway in colon cancer cells. *J. Cell. Physiol.* 225 266–275. 10.1002/jcp.22261 20509140

[B63] Yang Z. J. Chee C. E. Huang S. Sinicrope F. A. (2011). The role of autophagy in cancer: therapeutic implications. *Mol. Cancer Ther.* 10 1533–1541. 10.1158/1535-7163.mct-11-0047 21878654 PMC3170456

[B64] Zhao J. Lian Y. Lu C. Jing L. Yuan H. Peng S. (2012). Inhibitory effects of a bisbenzylisoquinline alkaloid dauricine on HERG potassium channels. *J. Ethnopharmacol.* 141 685–691. 10.1016/j.jep.2011.08.054 21920426

